# Spatial metrics in fire ecology: seeking consistency amidst complexity

**DOI:** 10.1002/brv.70140

**Published:** 2026-02-03

**Authors:** Alexander R. Carey, Geoffrey J. Cary, Teigan Cremona, Hugh F. Davies, Brett P. Murphy, Sam C. Banks

**Affiliations:** ^1^ Research Institute for the Environment and Livelihoods, Faculty of Science and Technology Charles Darwin University Casuarina NT 0810 Australia; ^2^ Fenner School of Environment & Society, The Australian National University Canberra ACT 2600 Australia; ^3^ School of Environmental and Rural Science, University of New England Armidale NSW 2350 Australia

**Keywords:** spatial metrics, fire ecology, fire regimes, ecological relevance, topic modelling

## Abstract

Technological advances, including remote sensing, have led to a proliferation of metrics used in ecological studies to examine spatial patterns of fire regimes and their ecological effects. Researchers can use many different metrics to analyse spatial variation in both fire events and resulting fire regimes, including fire size, shape, intensity, frequency and seasonality. However, variation in metric selection, definition, and application can yield inconsistent findings and/or difficulty in the synthesis of findings from different studies. This review aims to (*i*) visualise trends in spatial terminology within the broader fire ecology literature, (*ii*) characterise the variability among metrics for describing spatial fire patterns, and (*iii*) evaluate the ecological relevance of metrics, identifying opportunities to enhance consistency.

This review comprises three sections. First, we used topic modelling to determine topic trends in fire ecology over the last three decades (1991–2025). We found a shift from studies primarily focused on individual fire regime aspects to a more holistic approach incorporating multiple fire regime aspects, including spatial patterns. Second, we present findings from a qualitative review, revealing marked variation in metric selection within and among taxa, biomes, and the technique used to measure spatial metrics. We also identified ecological processes, such as dispersal capacity, that prompt researchers to use more specific metrics to analyse their study system more precisely, leading to less consistency among studies. Finally, we offer recommendations for enhancing metric consistency whilst maintaining the flexibility to adapt and develop those metrics most relevant and informative for a given objective.

As technological advances allow for a more complete description of the spatial attributes of a fire regime, there is a potential trade‐off between generality and precision, reducing comparability among studies. To ensure ecological relevance, it is crucial to consider the characteristics of data, landscape, and ecological contexts when selecting and applying metrics. Recent advances in landscape analysis techniques, such as through applying information theory, are leading to metrics that can be broadly applicable across study systems. Using the most generalised metrics possible, reporting standardised metrics of all fire regime components, aligning with landscape ecology where appropriate, and staying updated on emerging techniques will ensure the fire ecology field can move forward with a more coordinated approach.

## I. INTRODUCTION

As with any discipline, fire ecology is continually shaped by new findings, technologies, and concepts. The discipline has grown dramatically from the early 20th century, when total fire suppression was a dominant management paradigm, to acknowledgement in the 1960s of fire as a natural disturbance process driving ecological dynamics (Krebs *et al*., 2010). Since then, researchers have been seeking to understand the intricacies of fire regimes and their impacts on species and ecosystems to enable improved fire management for conservation and restoration.

The term ‘fire regime’ refers to the patterns and characteristics of fires in an ecosystem, primarily including the frequency, intensity, extent, and season of fires. The emergence of the ‘fire regime’ concept in the 1960s has shaped the trajectory of research in fire ecology (Christensen, 1993; Gill, 1975; Heinselman, 1981). The strict selection of the core elements of the fire regime (i.e. frequency, intensity, extent, season) is a necessary simplification of an incredibly complex process with numerous and diverse variables and, as such, there has been much flexibility in the way the fire regime concept has been applied in research (Krebs *et al*., 2010). This flexibility is a strength, allowing researchers to choose metrics of fire regime elements befitting specific questions, and the availability of resources and data. However, while technological advances have expanded the range of metrics available in fire ecology, the growing number of metrics with distinct definitions and applications also increases the potential for inconsistency. Moving the field forward in a clear and data‐informed way requires balancing the adaptability of these metrics with the need for a foundational level of consistency in metric definition and application (Westgate & Lindenmayer, 2017).

The spatial components of the fire regime, such as fire extent and patchiness, have been understudied in comparison to the temporal components, such as fire frequency or interval (Mason & Lashley, 2021; Williamson, Ellis & Bowman, 2022). Historically, this has been attributed to the difficulty in obtaining high‐quality, high‐resolution spatial data (Gill & Allan, 2008), the dominant research focus on plants of limited dispersal ability (Bowman, 2007), and the overall difficulty of designing and implementing studies of spatial components in the field at the necessary spatial scale (Lutz, Larson & Swanson, 2018). However, many of these limitations have faded in recent years with the emergence of new technologies, experimental approaches, and a broader taxonomic focus, encompassing a wide range of plants, animals, and ecosystems. For example, satellite remote sensing provides periodic large‐scale monitoring that allows researchers to map the extent of fires, their severity, which is related to fire intensity, and patchiness, and overlay the attributes of repeated fires to derive fire histories (Szpakowski & Jensen, 2019). By combining these fire data with measurements of population distribution and abundance, researchers can elucidate species' responses to different fire regimes (Keith, 2012).

**Fig. 1:**
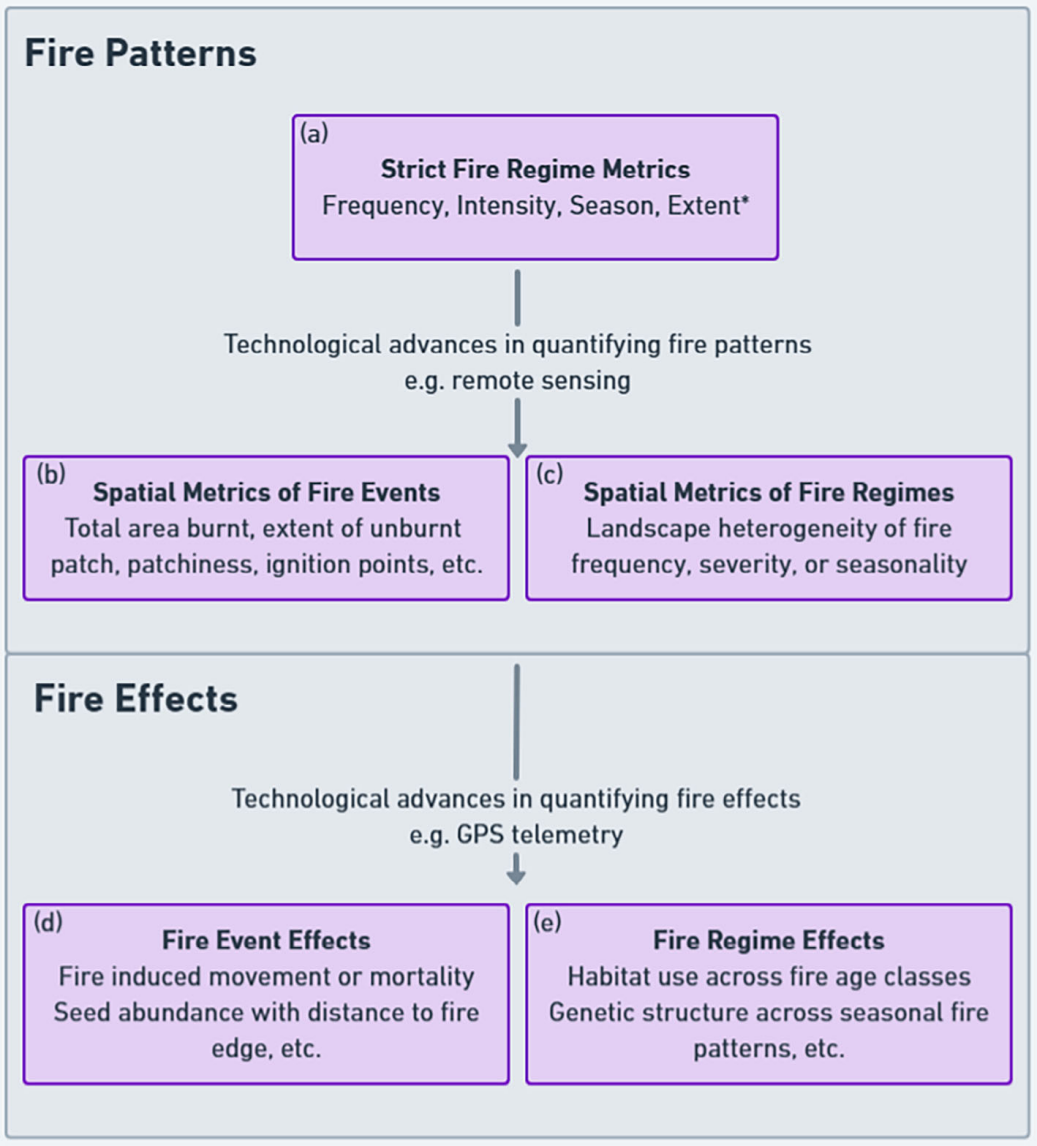
Flow diagram showing the increasing specificity of spatial metrics in fire ecology, allowing researchers the flexibility to apply metrics of high ecological relevance. *, added as a later inclusion to the strict definition of the fire regime (Gill & Allan, 2008). GPS, global positioning system.

Fire ecology uses information on the patterns of fire (e.g. spatial and temporal patterns of fire events or regimes) and on the responses of individuals, populations, species, or ecosystems to fire. Technological advances have not only improved methods for quantifying spatial fire patterns, but also the ability to measure the resulting ecological effects of fire. Early fire regime definitions were of a point‐based concept, where regime components (frequency, intensity and season) were measured at multiple points across the landscape (a in Fig. 1). Fire extent was not considered a component of the fire regime, in this context, but spatial heterogeneity in a fire regime could be used as a descriptor of spatial pattern (Gill, 1975). With technological advances facilitating greater quantification of spatial fire patterns, spatial metrics of fire events were applied and more commonly incorporated into definitions of the fire regime (Gill & Allan, 2008; b in Fig. 1). As discussed in Krebs *et al*. (2010), the definition of fire regime has been extended from the strict definition of frequency, intensity, extent, and season, to incorporate more complex, combined, or derived parameters (c in Fig. 1). Additionally, technological advances in the collection of ecological data have provided researchers with an array of metrics to explore the effects of fire across events and regimes (d and e in Fig. 1). Technological advances in wildlife tracking, population genetics, and simulations have opened new avenues for exploring the ecological effects of fire events and regimes with greater depth and precision and have led to the emergence of diverse spatial metrics that capture the less‐tangible ecological consequences for taxa with more spatially complex population dynamics.

There have been rapid advances in technologies for monitoring the fine‐scale movements of individuals and their populations. Satellite‐based tracking methods, including global positioning system (GPS) telemetry devices are increasingly used in fire ecology to track wildlife responses to fire events and in post‐fire landscapes (Berry *et al*., 2016; McGregor *et al*., 2014; Parker *et al*., 2021). The increasing affordability of these techniques means more individuals can be tracked, and improved accuracy provides high‐quality data on how species survive fire events, how they persist post‐fire, and how habitat and resource use are related to fire histories (Nimmo *et al*., 2019). Reductions in the size and weight of collars also means these techniques can be applied to a wider range of organisms such as small, highly mobile birds (Collett *et al*., 2023). Genetic techniques have also been increasingly applied to study population responses to fire and can provide information that is beyond the scope of other techniques (Driscoll *et al*., 2014; Steinitz *et al*., 2012). For example, genetic insights have been used to assess the influence of historical fire regimes on the phylogeography of plants (Bradbury *et al*., 2016) and to identify the mechanisms of recovery that allow animals to persist in fire‐prone landscapes (Banks *et al*., 2017b). Combining these fine‐ and broad‐scale techniques of population monitoring with different experimental designs has led to a diversity of approaches for applying spatial metrics in fire ecology studies.

To disentangle the interacting effects of fire regime components, sophisticated approaches must be used to identify population responses to spatial attributes of a fire regime. Simulation modelling is particularly useful for exploring complex fire effects at spatial and temporal scales too large to be implemented as a real‐world (*cf*. simulation) experiment. At a landscape scale, simulations have been effectively applied to explore fire‐ and climate‐driven changes to ecosystems (Lenihan *et al*., 2008) or the biodiversity consequences of contrasting management options (Davies *et al*., 2021, 2023). At a finer spatial scale, simulations have been used to examine dispersal and life‐history responses to fire in both plants (Dickman *et al*., 2023; Pausas & Lloret, 2007) and animals (Banks, Davies & Cary, 2017a; Banks *et al*., 2017b; Smith *et al*., 2016). Simulations can be strengthened by incorporating empirical data from natural and manipulated studies. Fire experiments are particularly useful as they can be more robust than correlative studies in demonstrating causation, albeit at a cost of realism or complexity (Andersen, 2021; Williams, Woinarski & Andersen, 2003).

The various technological advances outlined above have led to a proliferation in spatial metrics and the way they are selected, defined, and utilised to elucidate population and ecosystem change in response to fire regimes. Researchers can choose from among many metric options to analyse fire size, shape, and spatial metrics of the other fire regime components (frequency, intensity, and seasonality). Whilst this flexibility in the specification and application of spatial fire regime metrics can enable metrics to be tailored to individual systems and studies, it makes it difficult to identify emerging consensus in fire ecology research. Hence, the objective of this review is to (*i*) visualise trends in the use of spatial terminology within the broader fire ecology literature, (*ii*) characterise the variability among metrics (including their definitions and operational methods) used to describe the spatial patterns of fire events and regimes, and (*iii*) evaluate the ecological relevance of different metrics and, where possible, identify opportunities to enhance consistency between them. This review is organised into three sections. In Section II, we use topic modelling to explore the growth in complexity of spatial metrics in fire ecology. In Section III, we conduct a qualitative review characterising the variation of spatial metrics of fire patterns and uncovering the drivers of that variation. Finally, in Section IV, we make recommendations on ways to advance the field of fire ecology by applying metrics with greater consistency whilst maintaining the flexibility to apply the most appropriate metric to individual study systems.

## II. TRENDS IN FIRE ECOLOGY

### (1) Methods

#### (a) Data

Topic modelling is a statistical method used in text analysis to identify prevalent themes and topics in a large collection of documents. We conducted topic modelling to gain a broad overview of the common themes relating to fire regimes in the fire ecology literature, how they relate to each other, and how the frequency of use of spatial terminology has changed through time when compared to other fire regime components. To perform a thorough review that incorporates key components of the fire regime concept and the taxonomic diversity of the field, we searched the Clarivate Analytics *Web of Science* database of published articles (mainly peer‐reviewed academic journals). We searched for articles using the Boolean search terms: (ALL = (frequency OR intensity OR severity OR size OR extent OR pyrodiversity OR mosaic OR pattern)) AND (ALL = (animal OR plant OR biodiversity OR flora OR fauna OR vegetation OR wildlife)) AND (ALL = (fire OR burn*)). This search resulted in 41,080 articles which we then refined using the *Web of Science* categories, selecting fire‐ecology‐related fields (ecology, environmental sciences, forestry, plant sciences, biodiversity conservation, and remote sensing). This resulted in 22,698 articles which were further refined using the Citation Topics Meso field for relevant topics (forestry, zoology and animal ecology, soil science, remote sensing, entomology, environmental sciences, and phylogenetics and genomics) identifying 18,569 articles for further investigation. The final step within *Web of Science* was to filter out irrelevant topics from the Citation Topics Micro field (composting, fracking, and ocean colour).

We downloaded all 16,656 articles from this search that were published between 1990 and January 27, 2025. One of us (A.R.C.) manually checked the title and abstract of each article to ensure that only fire ecology studies were included in our analysis. We included studies exploring both fire patterns and fire effects (Fig. 1). A total of 8,826 articles from 1991 to 2025 met this criterion and were considered for further analysis (see online Supporting Information, Appendix S1). Despite the *Web of Science* search not producing any studies before 1990, we chose not to supplement the search with other methods due to the possibility of confounding effects and the diminishing returns in numbers of papers prior to 1990 (Fig. S1).

We used the function ‘make_dtm’ within the ‘*revtools’* R package (Westgate, 2019) to combine the title, abstract and keywords of each article to form the dataset (‘corpus’) and removed meaningless terms (i.e. ‘stop words’, for example, *the*, *or*, *and*, *which*), numbers and punctuation. The remaining words were stemmed (reduced to their root form, for example, *species* and *speciate* became *speci*) and tested for bi‐grams (pairs of words that retain semantic information that would be lost if the words were analysed separately, e.g. White House).

#### (b) Topics

We used Latent Dirichlet Allocation (LDA) modelling to identify common topics reported in our data set. In this context, a topic is a set of co‐occurring words that helps to describe the content of different articles in a corpus (Murakami *et al*., 2017). Importantly, LDA is an unsupervised method, meaning the categories emerge using an objective methodology applied to the corpus, rather than being predetermined by a human evaluator. The LDA model identifies sets of co‐occurring words that are more frequently presented within the same linguistic context than expected by chance alone (Luiz *et al*., 2019). These co‐occurring words tend to purport a similar meaning and refer to a similar subject, thus allowing topics to be defined. To select the number of topics we used the *ldatuning* R package (Nikita, 2016) and created 50 different LDA models by varying the *K*‐parameter from 1 to 50. The number of topics in our LDA model was selected using the optimisation method proposed by Deveaud, SanJuan & Bellot (2014) to evaluate the optimum number of topics (Fig. S2). Using this method, we identified 20 topics within the selected fire ecology corpus and chose a topic name that represented the top words and articles in each topic (Table 1). The probability of each topic across the entire corpus and the number of documents to which each topic is assigned as primary is shown in Table S1.

**Table 1:** Uncovered topics from 8826 research articles about fire ecology published during the period 1991–January 2025.

Topic no.	Topic name	Topic words (stemmed)
1	Animal ecology	habitat, popul, rang, effect, select, size, nest, resourc, declin, individu, impact, surviv, studi, avail, conserv, larg, respons, forag, affect, anim
2	Climate change	wildfir, increas, climat, climat_chang, region, impact, activ, drought, chang, futur, larg, global, emiss, trend, potenti, extrem, warm, vulner, condit, expect
3	Disturbance	disturb, interact, chang, ecosystem, effect, resili, influenc, dynam, function, role, alter, import, process, understand, direct, affect, respons, result, structur, herbivori
4	Ecological response	speci, site, commun, abund, rich, divers, bird, differ, respons, composit, sampl, group, studi, effect, found, diversity, speci_composit, taxa, similar, assemblag
5	Fire prediction	model, predict, risk, simul, probabl, base, develop, assess, potenti, data, spread, scenario, ignit, estim, variabl, wind, result, paramet, evalu, valu
6	Forestry	pine, forest, stand, regener, site, restor, ponderosa, mountain, harvest, conif, structur, high‐sever, longleaf, forest_structur, establish, snag, condit, understori, domin, similar
7	Fuel treatment	fuel, treatment, prescrib, effect, reduc, load, intens, flammabl, reduct, thin, behavior, potenti, surfac, control, litter, behaviour, fine, intensity, manag, hazard
8	Grassland	plot, grassland, cover, increas, graze, shrub, effect, grass, control, nativ, restor, experi, reduc, invas, forb, exot, decreas, domin, greater, result
9	Management	manag, research, ecolog, land, conserv, provid, plan, includ, develop, biodivers, tool, understand, protect, identifi, inform, impact, requir, focus, knowledg, system
10	Plant ecology	seed, plant, speci, germin, resprout, heat, bank, trait, leaf, seedl, high, degre, recruit, differ, flower, persist, life, dispers, produc, smoke
11	Post‐fire recovery	year, time, post‐fir, sinc, recoveri, success, follow, chang, postfir, studi, rate, show, similar, decreas, increas, recov, initi, short, dynam, post
12	Regional analysis	area, forest, studi, land, occurr, region, show, main, zone, high, result, analyz, affect, analysi, factor, cover, event, southern, locat, fires
13	Remote sensing	data, method, remot, detect, sens, assess, index, imag, estim, indic, base, landsat, area, ndvi, valu, monitor, analysi, modis, deriv, ratio
14	Season	season, dure, burnt, period, year, product, month, grow, annual, summer, occur, number, spring, averag, late, earli, total, activ, winter, differ
15	Severity	burn, sever, area, high, unburn, moder, result, effect, evalu, assess, compar, higher, upland, composit, pre‐fir, post‐burn, recent, unburn_areas, indic, ratio
16	Soil ecology	soil, carbon, loss, effect, water, properti, temperatur, biomass, nutrient, increas, moistur, total, depth, eros, impact, studi, surfac, site, concentr, measur
17	Spatial	landscap, pattern, variabl, variat, patch, larg, relationship, size, scale, vari, spatial, relat, influenc, elev, heterogen, distribut, topography, explain, spatial_pattern, determin
18	Temporal	regim, frequenc, histori, histor, frequent, park, interv, suppress, nation, record, rang, suggest, past, recent, current, associ, period, occur, chang, centuri
19	Tree dynamics	tree, densiti, mortal, growth, canopi, size, stem, height, surviv, bark, rate, seedl, damag, larg, recruit, basal, measur, increas, diamet, sapl
20	Vegetation	veget, type, savanna, differ, woodland, high, shrubland, distribut, result, relat, open, associ, rainfal, determin, domin, transit, structur, environ, australia, biom

The LDA model calculates the probability of each topic within a document (gamma), which allows each document to be assigned its most probable topic. To determine trends in topic prevalence, we calculated the proportion of papers that were allocated to each topic in each year. To do so, we fitted binomial generalised linear models (GLMs) for each topic, with proportion of papers as the response variable, and year as the explanatory variable.

The LDA model generates a matrix containing the weight (i.e. probability of occurrence) of each word within each topic, which can then be summarised using an association (dissimilarity) metric and subjected to multivariate ordination analysis. Using the *vegan* R package (Oksanen *et al*., 2013) we calculated the Bray–Curtis distance between each pair of topics using a matrix of the weights of each word within each topic (‘word’ distance matrix) to investigate topic similarity. We used non‐metric multidimensional scaling (NMDS) to visualise the distance matrix representing the semantic variation between topics.

### (2) Results

**Fig. 2:**
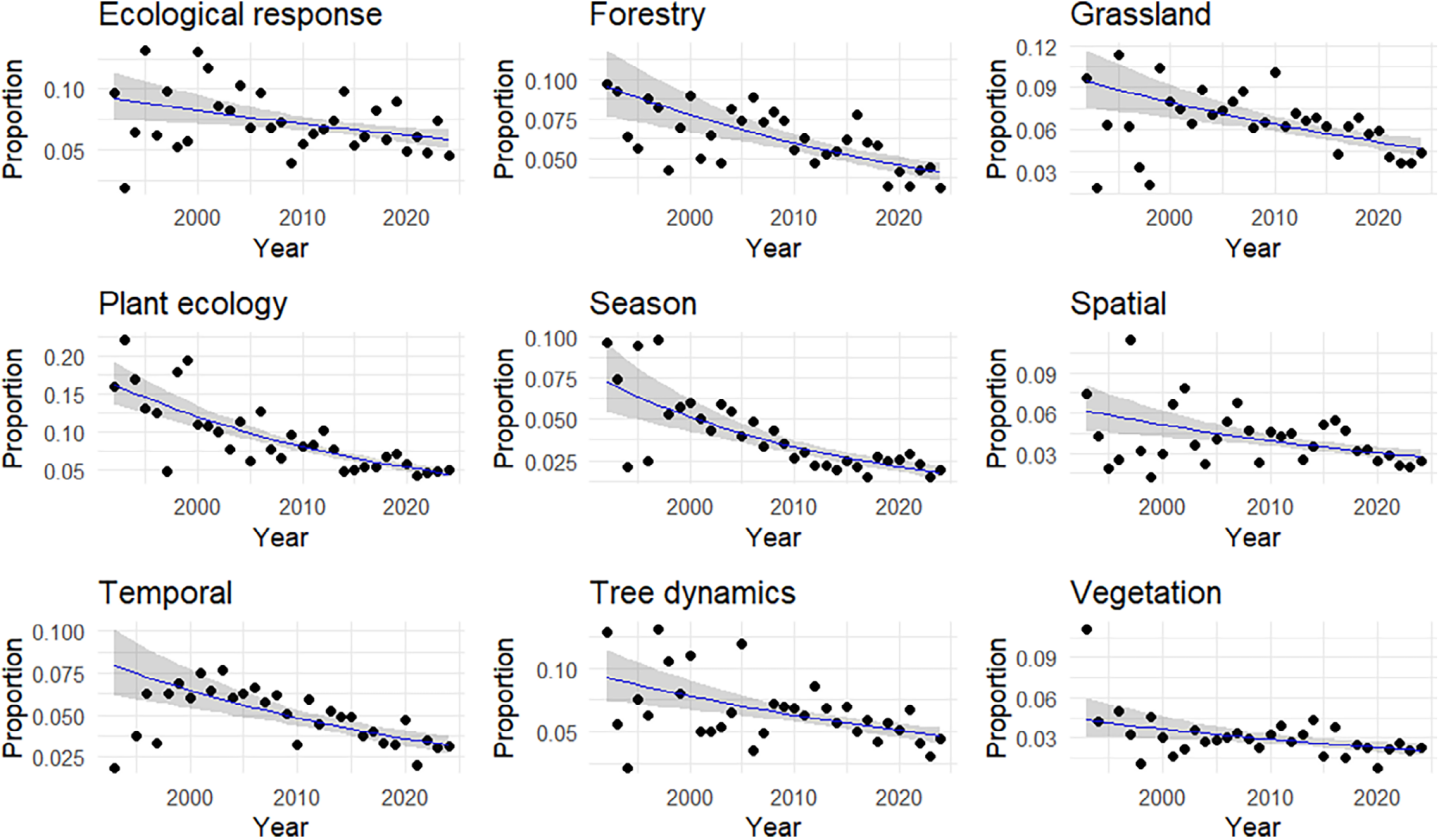
The proportion of total studies in each year allocated to each topic as the dominant topic, for all topics that significantly decreased in popularity between 1991 and 2025, with fitted generalised linear models (GLMs; blue lines) and 95% confidence intervals (shaded area).

We found that nine topics declined in prevalence between 1991 and 2025, six topics increased in prevalence, and five topics remained stable (Table S2). The topics that declined in prevalence included many of the topics describing individual components of fire regimes (e.g. spatial, temporal, season), as well as traditionally point‐based topics or location‐specific topics (e.g. forestry, plant ecology, tree dynamics; Fig. 2).

**Fig. 3:**
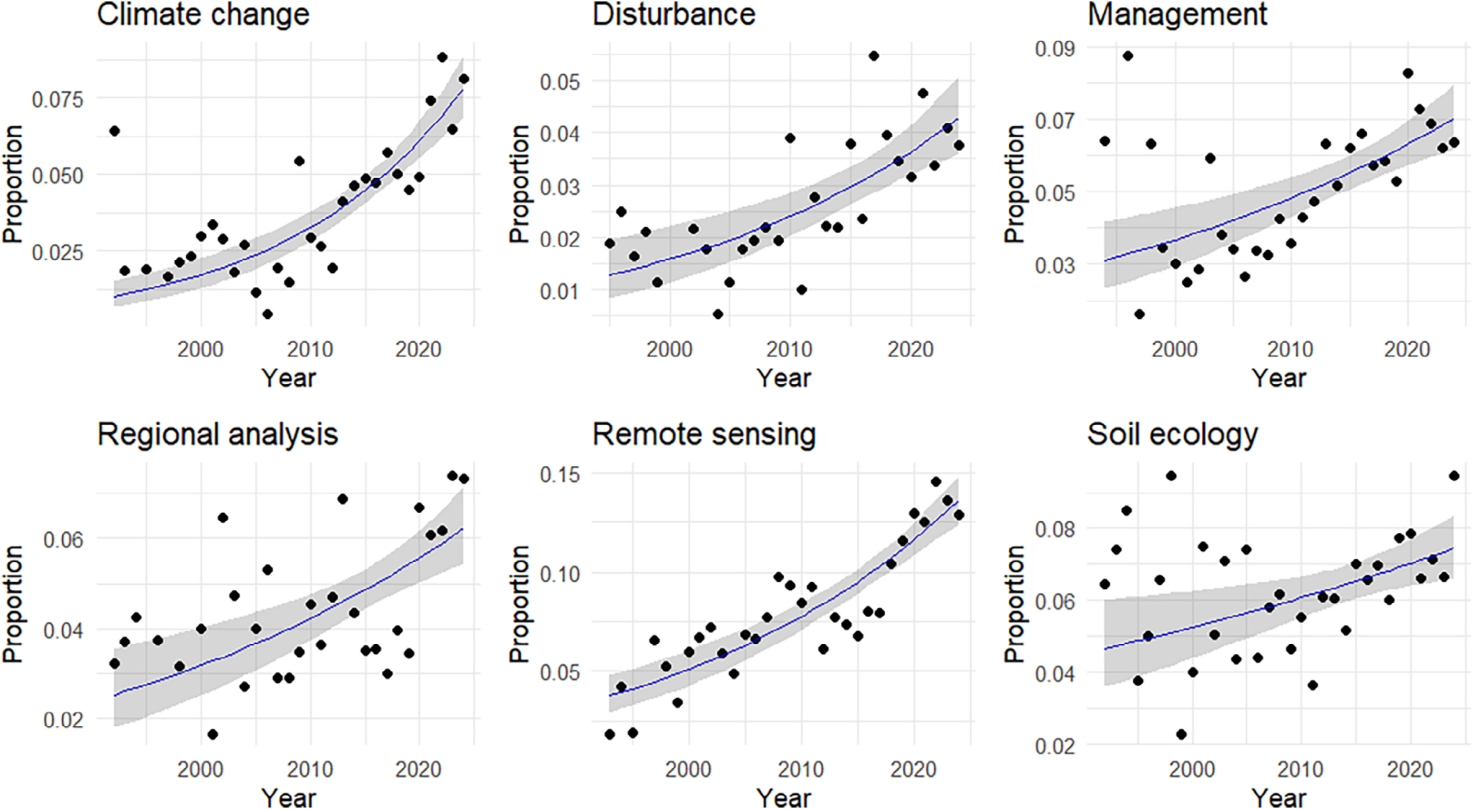
The proportion of studies in each year that were allocated to each topic as the dominant topic, for all topics that significantly increased in popularity between 1991 and 2025, with fitted generalised linear models (GLMs; blue lines) and 95% confidence intervals (shaded area).

The topics that increased in prevalence included topics that represent a mix of fire regime components (e.g. disturbance, climate change, management) and/or utilised broad spatial analytical techniques (e.g. remote sensing, regional analysis) (Fig. 3). Overall, this does not suggest that the declining topics were less published than the increasing topics (Fig. S3), but reflects that fire regime themes are transitioning from a once time‐specific and point‐based field to a more holistic spatiotemporal field. All topic trends with fitted GLMs and generalised additive models (GAMs) are shown in Fig. S4.

**Fig. 4:**
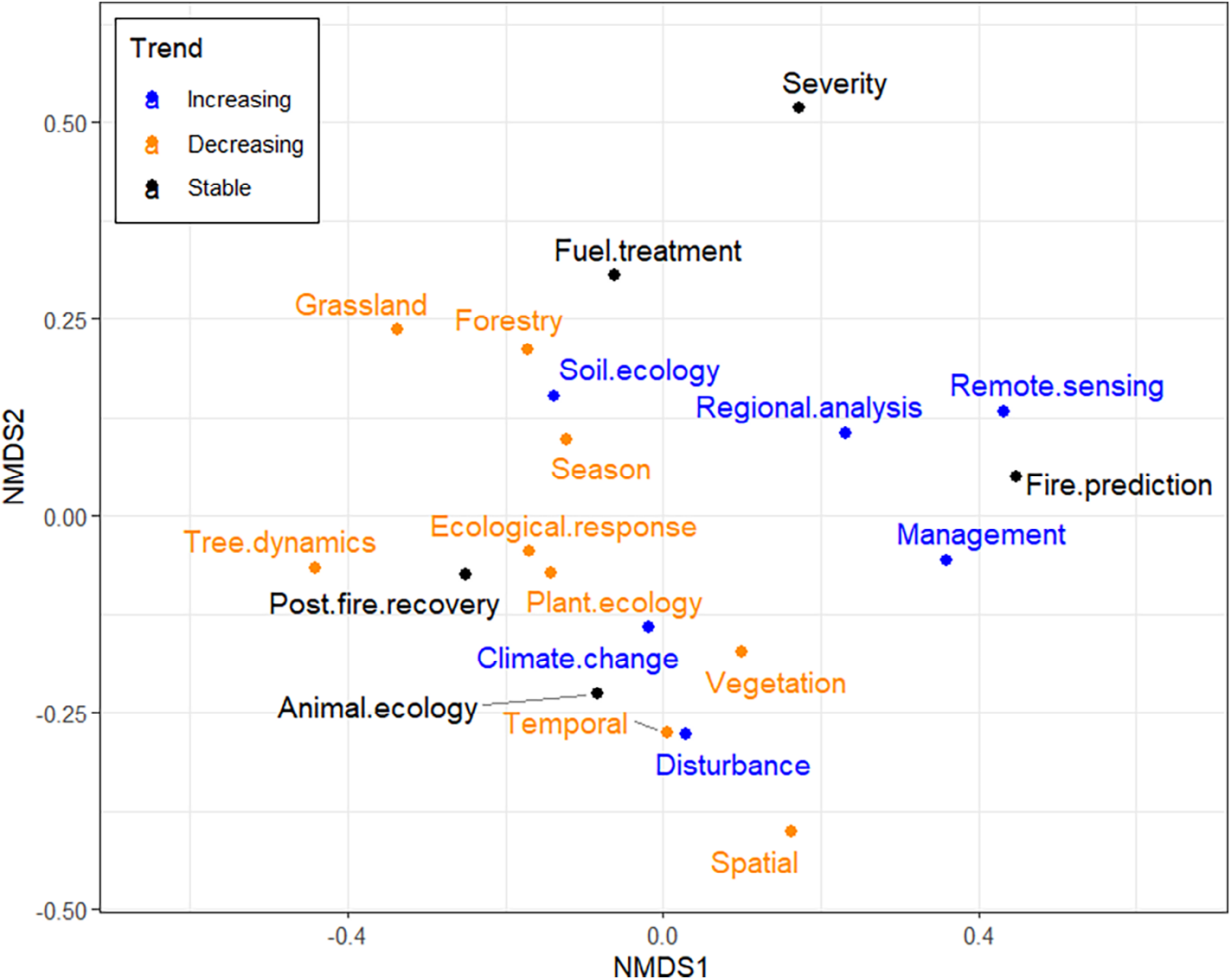
Non‐metric multidimensional scaling (NMDS) plot of Bray–Curtis dissimilarity matrix between topics from the fire ecology literature obtained from topic modelling. Five dimensions were analysed to balance data fit and interpretability. The plot shows the distribution of topics in two dimensions, with the distance between the topics reflecting the dissimilarity in their word composition. The stress value of the NMDS plot is 0.07, indicating a very good fit of the plot to the original dissimilarity matrix. The colours indicate the trend in topic prevalence.

Topics that were close together on the NMDS plot were more similar in word usage and semantic content (e.g. temporal and disturbance) than topics that were far apart (e.g. severity and spatial; Fig. 4). We chose five dimensions with a stress value of 0.07, which indicates a very good representation of the variation between topics, with values <0.2 indicating a good fit and >0.3 indicating a poor fit (Clarke, 1993). The NMDS1 axis mainly separates topics on the left (e.g. tree dynamics), which are well‐established topics with minimal impact from recent technological advances, from those on the right (e.g. remote sensing), which are complex multidimensional topics that have emerged as key interests over the last three decades aided by technologies such as remote sensing. The NMDS2 axis highlights the separation between topics focused on controlling fire, particularly reducing high fire severity, and those examining ecological processes and specific ecological elements, such as spatial and temporal patterns.

The topic modelling results highlight a broad shift in fire ecology research, from a primarily temporal and point‐based focus towards a more integrated spatiotemporal approach. This increasing complexity raises important questions about how spatial metrics are being selected, defined, and applied across studies. To explore this further, we conducted a qualitative review examining the variability in spatial metric use for analysing fire regime and fire event effects and investigated the ecological drivers behind metric specification.

## III. VARIABILITY IN THE USE OF SPATIAL METRICS IN FIRE ECOLOGY

We conducted a qualitative review of fire ecology studies that actively applied spatial metrics to investigate fire patterns and their resulting ecological effects. We reviewed a subset of studies from the broader topic modelling search described in Section II that explicitly analysed spatial fire attributes rather than mentioning spatial variation descriptively. These 606 studies were supplemented by studies from a more directed search that produced 21,999 studies (see legend to Table S3). We then screened the titles and abstracts for relevance to both spatial fire metrics and their ecological effects, resulting in 930 articles for qualitative review (Appendix S1).

Due to the shift towards more spatially complex approaches identified through topic modelling, it is critical to understand how spatial metrics are currently selected, defined, and applied across the fire ecology literature. This understanding can highlight both the opportunities and challenges for improving consistency and ecological relevance in future research. First, we characterise the variability among the different types of metrics used to describe spatial fire patterns. Second, we explore how these metrics are applied to assess fire regime and fire event effects and examine the ecological processes that drive variation in metric specification.

### (1) Metrics of fire patterns

#### (a) What metrics are being used to explore spatial fire patterns?

To interpret the selection and use of spatial metrics of fire patterns, we grouped relevant terms into broad metric categories (Table 2). Breaking down the spatial component of the fire regime into its constituent metrics opens a wide range of possibilities and presents a significant challenge. Early definitions of the fire regime used a point‐based concept. The spatial component was not originally considered in the fire regime concept partly due to the view that it was more relevant to particular effects and not universally important to regimes (Gill, 1975; Gill & Allan, 2008). Subsequent definitions included a component for size or area and recognised that variations of patchiness can be applied to specific systems (Christensen, 1993; Gill & Allan, 2008; Heinselman, 1981). Li, Corns & Yang (1999) demonstrated that inter‐fire interval (and hence fire frequency) is mathematically related to fire size, as larger fires cover more of the landscape and disproportionally impact mean fire interval. Therefore, interval can be used broadly and deconstructed into its fire‐area‐related parts as suited to the system in question. Theoretically, researchers choose a spatial metric that is relevant to the dispersal capacity, life history, or a previously established interaction between fire and their study system. In making this choice, an available selection of metrics from the literature helped to provide guidance. In evaluating papers that analysed or discussed spatial metrics, we selected a set of broad spatial metric categories to summarise. For example, Krebs *et al*. (2010, p. 63) listed the spatial components as “Extent, Fire size, Shape of fire, Ignition points, Area burned per decade…”. We modified this list by combining extent and fire size, converting area burned per decade to ‘Spatiotemporal’, and adding spatial severity and spatial seasonality to encompass all the metrics we found in this review (Table 2). Each study generally employed one broad spatial metric category. However, it was not uncommon to have two, and on occasion, when the spatial aspect of fire was the key focus, three and four broad spatial metric categories (hereafter referred to more simply as metric groups). The metrics examined here incorporate both fire regime effects (typically used in studies of species and community dynamics at broad‐scale distributions) and fire event effects (typically used in autecological studies of fine‐scale responses). The variation between these metric levels is explored in Section III.2.

**Table 2:** Broad classes of spatial metrics used in fire ecology studies.

Broad class of spatial metric	Definition	Example metrics	Example studies
Fire size	Metrics that delineate the boundaries around or area inside of a fire event	Total area burnt; extent or perimeter of burnt or unburnt area	Turner *et al*. (1997); Lees *et al*. (2022)
Fire shape	Metrics that define the shape of a fire event or specific component of a fire event	Patchiness; distance to burnt or unburnt boundary	Tasker *et al*. (2011); Rocha *et al*. (2022)
Spatiotemporal	Metrics that quantify the spatial distribution of fires through time	Diversity of fire ages in the landscape; extent of long unburnt patches	Tepley & Veblen (2015); Robertson *et al*. (2022)
Spatial severity	Metrics that quantify the spatial distribution of fire severity	Area burnt at low, moderate, and/or high severity	Berry *et al*. (2016); Stillman *et al*. (2021)
Spatial seasonality	Metrics that quantify the spatial distribution of fire seasonality	Distribution of fire events throughout the year or in a particular season	Radford *et al*. (2021); Govender *et al*. (2006)
Ignition points	Metrics that quantify the spatial distribution of ignition points	Density of ignition points (e.g. number of ignitions per km^2^)	Bradstock *et al*. (2006)

#### (b) Are spatial metrics of fire patterns used variably?

Most studies exploring fire patterns and/or their ecological effects come from the USA, and Australia, with other notable contributions from Mediterranean countries, China, Canada, and Brazil (Table S3). A wide range of biomes were studied with a predominant focus on temperate broadleaf and montane forests (e.g. from the western USA and eastern Australia), savannas (e.g. from Africa, Brazil, and northern Australia), and mediterranean vegetation (e.g. from southwestern Australia and the Mediterranean). All broad classes of spatial metrics of fire patterns were used across all biomes, however, some biomes may foster the use of certain metrics more than others. For example, biomes with strong seasonality in rainfall, such as savannas, often utilise spatial metrics of fire seasonality (Radford *et al*., 2021). Likewise, metrics of spatial severity are particularly common in biomes that experience low‐frequency fire, such as temperate forests, and generate patterns of mixed severity (Halofsky *et al*., 2011).

With a direct interaction with fire, plants were the most well‐represented taxa in studies of fire patterns (283 out of the 930 studies reviewed here) utilising all broad classes of spatial metrics. However, when examining ecological effects, many plant studies measured distance from seed source and therefore fire shape metrics were commonly used (Coop & Schoettle, 2009). Mammals (226 of 930) and birds (228 of 930) were also well‐represented taxa in studies examining ecological effects of fire patterns, whilst invertebrates (69 of 930), and reptiles and amphibians (60 of 930) remained poorly represented taxa. Some studies measured fire effects across multiple taxa (48 of 930) providing useful comparisons for broadscale management. Most studies were of short duration (1–3 years), but some studies were long‐term (e.g. up to 37 years; Kashian *et al*., 2012). Studies either focused on a specific fire event, or across a fire regime, with some studies combining both these temporal scales (Fontaine & Kennedy, 2012; Kiss & Magnin, 2003). Most studies used a single spatial metric, however, some studies used multiple metrics (up to eight; Radford *et al*., 2015).

The methods used to characterise spatial attributes of the fire were dominated by remote‐sensing techniques, particularly satellite‐based techniques, which are now readily available and affordable to researchers and can be used to describe fine‐scale (e.g. Landsat can provide a 30 m spatial resolution) fire patterns and fire histories over large areas (Table 3). On‐ground methods were popular for studies reconstructing historical fires, and experimental and prescribed fires were generally used when researchers sampled populations before and after fire to understand the response to, and recovery from, individual fire events. Simulations allowed researchers to explore questions beyond the scope of practical limitations but often incorporated data from other techniques, such as remote sensing. For some studies, it was unclear what technique was employed, making interpretations difficult without understanding the relevance of the technique, the scale utilised, and any other considerations.

**Table 3:** Common techniques used to measure spatial patterns of fire and examine the ecological effects with examples of metrics used and methods applied.

Technique	Example metric	Example method	Example study
Remote sensing: satellite	Pyrodiversity: heterogeneity in burn severity	Burn severity, as measured by change in per cent canopy cover based on the satellite‐derived, relativised differenced normalised burn ratio (dNBR) score	Tingley *et al*. (2016)
Remote sensing: aerial imagery	Distance to nearest edge of lower severity	Aerial photography; areas with all trees killed were labelled high severity; on‐ground truthing; FRAGSTATS to calculate Euclidean distance (m) from each point (i.e. 10‐m grid cell) within a high‐severity patch to the nearest edge of lower severity	Haire & McGarigal (2010)
On ground	Heterogeneity in burn severity	Five parallel 100 m‐long belt transects at 20 m intervals; assessed patchiness of fire severity by measuring the proportion of burned stems and individuals; used a runs test in the software *Past 2.15* where a random distribution indicates more heterogeneous distribution of fire severity	Dodonov *et al*. (2014)
Simulation	Simulated fire size	Simulation; graphically analysed the frequency distribution of fire sizes from the records of all fire events separately for the four combinations of topographic roughness and weather variability scenarios. This metric was not directly modelled on population impact but is related to the spatial heterogeneity in fire frequency and the number of refuges present	Banks *et al*. (2017a)
Experimental/ prescribed	Fire pattern: presence of refugia (thickets)	Experimental burn that burnt 13 ha, removing the grass layer but leaving unburnt thickets	Yarnell *et al*. (2008)

### (2) Metrics of fire effects

The reasons behind the variation in use of spatial metrics highlighted in Section III.1.*b* are complex and varied. We now look more in‐depth at the way the spatial component of the fire regime is incorporated into analyses and at the ecological drivers of variation in the way metrics are selected and applied. We first provide a broad‐level look at spatial metric use for analysing fire regime effects *versus* fire event effects, and then a fine‐scale assessment of the ecological drivers of metric specification.

#### (a) How are spatial metrics being used to explore fire regime and event effects?

Variation in the selection, definition, and application of spatial metrics was primarily driven by the research questions and the system being explored: to achieve greater ecological meaning, researchers increased the specificity of the metric. This proliferation in metrics has also been observed in the landscape ecology literature (Costanza *et al*., 2019; Gustafson, 2019) and we discuss the implications of this in Section IV. Therefore, much of the variation in the use of metrics within taxa and biomes is by default driven by the increased ability to explore fire effects to suit the objectives of each study. For example, in exploring the concept of how pyrodiversity relates to species diversity, researchers combined ecologically relevant data with metrics of diversity of post‐fire age classes (Kelly *et al*., 2012; Taylor *et al*., 2012) or metrics of diversity of burn severity (Tingley *et al*., 2016) depending on the specific objectives and requirements of the study system.

Variation in the application of spatial metrics was particularly evident when comparing studies conducted at different spatial or temporal scales. Studies assessing fire regime effects often used broad‐scale metrics that quantified fire effects over multiple fire events, whereas studies examining fire event effects, often autecological in focus, tended to use fine‐scale metrics to capture responses to individual fire events. Such variation in metric application was expected as a necessary requirement to address differing study objectives adequately. Metrics of fire size and fire severity provided clear examples of how metric use can differ depending on whether the focus is on fire regimes or individual fire events.

Fire size metrics used to assess fire regime effects were typically applied in broader, community‐level studies using Landsat or MODIS (Moderate Resolution Imaging Spectroradiometer) satellite data to map the fire perimeter and examine broad patterns of distribution, abundance, or diversity (Farnsworth *et al*., 2014; Wilkin *et al*., 2021). Fire regime metrics were also obtained from on‐ground methods to reconstruct historical fires and understand long‐term forest dynamics (Floyd *et al*., 2000; Kennard & Moore, 2013; Kitchen, 2012; Tepley & Veblen, 2015), or simulated fire size to explore management approaches or long‐term demographics (e.g. Banks *et al*., 2017a; Bradstock, Bedward & Cohn, 2006).

For spatial severity metrics, studies that applied metrics of fire regime effects used satellite‐derived classifications of the landscape into severity classes that were then analysed independently or as a pyrodiversity metric (typically the Shannon or Simpson indices; Stillman *et al*., 2021). When applied at this broad scale, spatial severity metrics are typically calculated using the differenced Normalised Burn Ratio (dNBR). The dNBR compares the difference between pre‐fire and post‐fire near‐infrared and mid‐infrared bands from satellite (usually Landsat) images (Lutz *et al*., 2011). The dNBR values generated, usually between −0.6 and 1.2 in natural landscapes, are then stratified into four burn severity categories (high, moderate, low, and no detected change) and subsequently analysed (Lutz *et al*., 2011). Inconsistency can arise from this approach by classifying a continuous variable into discrete bins, resulting in some information inevitably being lost (Lutz *et al*., 2011). It has been recommended that researchers conduct ground‐truthing surveys to verify the dNBR results and ensure the severity classes are relevant to each vegetation type and the ecological process in question (Gale & Cary, 2022; Lutz *et al*., 2011).

One prominent issue is the ecological relevance of fire severity metrics and their relation to metrics of fire intensity (Han *et al*., 2021). For example, dNBR has been found to correlate strongly with pre‐fire vegetation height, suggesting that the vertical distance between flames and the top of vegetation will influence the severity category regardless of relevance to the studied taxa (Gale & Cary, 2022). Extended versions of the dNBR include the relative differenced normalised burn ratio (RdNBR) and the relative burn ratio (RBR), which can account for the amount of pre‐fire vegetation (Miller & Thode, 2007; Parks, Dillon & Miller, 2014).

In comparison, metrics of fire event effects are often more specific to the taxa and biome of interest. Fire size metrics of fire event effects included studies that conducted experimental burns to unpack fine‐scale species responses to fire, habitat use, and survivorship (Leahy *et al*., 2016; Vernes & Pope, 2001; Yarnell *et al*., 2008). Studies that applied spatial severity metrics of fire event effects used on‐ground methods to determine localised fire severity. These studies defined fire severity based on observable features relevant to their taxa (e.g. for invertebrates, proportion of leaf litter consumed; Huebner, Lindo & Lechowicz, 2012). It is necessary to delve deeper into the patterns that drive this fine‐scale variation to identify the common ecological reasoning and whether greater consistency can be achieved.

#### (b) What are the ecological processes driving metric specification?

With technological advances in remote sensing increasing both the spatial and temporal resolution of fire data, the possibility of applying metrics of greater specificity to research questions becomes achievable. Studies examined in this review have combined this improved fire data with data on spatial population dynamics obtained through satellite and GPS telemetry, genetics, fire experiments and simulations to ensure their metric choice is of ecological relevance. This proliferation of metrics, and methods to apply them, has the potential to produce a confusing literature with individual studies that are difficult to compare. However, by choosing the metric based on clearly defined objectives, guided by the scale and nature of the ecological process of interest (Gustafson, 2019), fire ecologists can ensure we are progressing in a process‐oriented way (Table 4). Here, we describe some of the ecological reasoning behind metric specification, with examples from common taxa, grouped by broad spatial metric categories.

**Table 4:** Examples of spatial metrics chosen in fire ecology studies, with the objective, scale and ecological considerations to provide context.

Objective	Scale	Relevant traits	Metric choice	Metric source	Reference
Test the hypothesis that pyrodiversity enhances reptile diversity	Landscape/Regime	Shelter preference; commonness	Extent recently burnt, extent long unburnt, fire age diversity	Satellite imagery: GIS; FRAGSTATS	Farnsworth *et al*. (2014)
Examine landscape‐level fire extent and severity on bird species responses	Landscape/Event	Dispersal ability; stem density	Total area burnt, area burnt at moderate severity and high severity	On‐ground surveys; government maps; GIS	Lindenmayer *et al*. (2014)
Assess whether changes in species richness and relative abundance could be predicted by fire pattern	Landscape/Event	Association with spinifex vegetation; dispersal ability	Conditional entropy	Experimental fire; satellite imagery; ‘landscape metrics’ (R package)	Doherty *et al*. (2024)
Evaluate seed abundance and species richness through seed dispersal at fire edges	Site/Event	Dispersal mechanism: animal, wind, explosive	Distance to edge	Satellite imagery, GIS; on‐ground measurements	Rocha *et al*. (2022)
Test whether species in spatially structured populations exhibit dispersal strategies to track resources through space and time	Landscape/Regime	Differential habitat preferences between adults and juveniles	Distance to edge, distance to nearest burn, fire size, spatial time since fire	Online government databases; on‐ground measurements and telemetry data	Stillman *et al*. (2022)
Investigate the processes and patterns of population recovery of two small mammals after fire	Site/Event	Sex‐biased dispersal; species‐specific demographic rates	Burnt or unburnt, distance to edge	Government maps; GIS; on‐ground validation	Banks *et al*. (2017b)
Investigate the landscape‐level drivers of reptile distributions	Landscape/Regime	Association with spinifex vegetation	Extent recently burnt, extent long unburnt, fire age diversity, extent of all fire age classes	Satellite imagery; GIS; FRAGSTATS	Nimmo *et al*. (2013)
Investigate how persistence may be sensitive to habitat patch dynamics in a simulated landscape setting	Landscape/Regime	Life‐history; dispersal; territory habitability	Rate of ignition (fire size), spatial pattern of ignition	Simulations	Bradstock *et al*. (2005)
Examine the factors that influence the distribution of termites	Site and landscape/Regime	Low mobility; strong habitat association with resources impacted by fire	Extent recently burnt, extent long unburnt, fire age diversity	Land management agencies; local knowledge; GIS	Avitabile *et al*. (2015)

GIS, Geographic Information Systems.

##### (i) Fire size

As described in Section III.2.*a*, fire size metrics have been applied at a range of spatial scales depending on the study objectives. For mammals, this often involved examining short‐term post‐fire responses such as habitat use (Lees *et al*., 2022). Some studies utilised pre‐fire information on home range size, movement patterns, resource availability, and predation risk to be able to understand post‐fire responses (Fordyce *et al*., 2016; Leahy *et al*., 2016; Yarnell *et al*., 2008). Fire perimeter and extent of burnt and unburnt patches were commonly combined with metrics of fire shape, severity, and frequency as well as habitat data to examine if mammals exhibited preferences for specific microhabitats or adjusted their patterns of resource use following fire. There is evidence for variable responses to these fire attributes due to predation pressure (Leahy *et al*., 2016), sociality and territoriality (Lappan *et al*., 2021), sex‐biased dispersal (Banks *et al*., 2017b), or specific habitat features (Delaney, Di Stefano & Sitters, 2021; Fordyce *et al*., 2016; Hulton VanTassel, Barrows & Anderson, 2015). An understanding of species pre‐fire context is therefore essential when considering metric selection and application and the covariates to examine alongside them (Table 4).

##### (ii) Fire shape metrics

There are many metric options for analysing how the shape of fire affects species responses. Studies in this review examined distance to unburnt areas (Rocha *et al*., 2022), the mean size of patches (Blakey *et al*., 2021; Legge *et al*., 2008), the density of patches (Haire, Coop & Miller, 2017; Kashian *et al*., 2012), and the diversity of patch shapes (Bradstock *et al*., 1996; Burgess & Maron, 2016). The software FRAGSTATS is particularly useful for analysing fire shape metrics and includes complex analyses of landscape configurations (McGarigal, 1995). For example, the ‘related circumscribing circle’ is a measure of fire‐mediated patch shape complexity based on the ratio of patch area to the area of the smallest circumscribing circle (Burgess & Maron, 2016; McGarigal, 1995). Plant studies often used fire shape metrics in combination with fire size metrics to examine patterns of recruitment, regeneration, and succession (Turner *et al*., 1997; Wan *et al*., 2014). Important variables documented include herbivory (Wan *et al*., 2014), serotiny and fire sensitivity (Bradstock *et al*., 2006; Robertson, Platt & Faires, 2019; Trauernicht *et al*., 2015), and landscape context variables such as rainfall patterns (Kelly *et al*., 2017; Reilly *et al*., 2017).

##### (iii) Spatiotemporal metrics

At a certain point in the landscape, the availability of key resources is determined by a range of factors including fire history (Haslem *et al*., 2011). However, spatial heterogeneity in fire frequency or the spatial extent of different fire age classes provides this information at a landscape scale (Nimmo *et al*., 2013). Spatial heterogeneity in fire frequency depends on the length and scale of observation but can be informative across studies and regions (Carey *et al*., 2025). Spatial extent of time since last fire metrics either focused on the proportion of a vegetation age class that was given *a‐priori* importance to the species or examined the diversity of fire age classes. *A‐priori* importance was most commonly given to extent recently burnt (Farnsworth *et al*., 2014), followed by extent long‐unburnt (Burgess & Maron, 2016) and then extent mid‐range burnt (Kelly *et al*., 2012), however, multiple ages were usually compared. Studies on multiple species found that the individual responses reflected species spatial requirements and ecological traits. For example, Nimmo *et al*. (2013) found that reptile occurrence was associated with mid‐successional vegetation as this age class coincides with a peak in spinifex (*Triodia* spp.) cover – a keystone structure for reptiles in semiarid and arid Australia. Other studies on reptiles highlighted the thermal properties of landscapes, including vegetation structure and composition, and how they influence reptile thermoregulation and provide thermal refuges (Robertson *et al*., 2022; Smith, Bull & Driscoll, 2012).

Metrics examining the diversity of fire age classes were often used to test the pyrodiversity hypothesis (Radford *et al*., 2021; Taylor *et al*., 2012). These metrics were used to test whether the diversity of fire ages was of benefit to either individual species or species assemblages (Kelly *et al*., 2012; Nimmo *et al*., 2013). There are several metrics available to summarise landscape heterogeneity; most commonly the Shannon index through FRAGSTATS (Avitabile *et al*., 2015; Delaney *et al*., 2021). Functional diversity metrics are increasingly used in fire ecology to quantify the multidimensional variability of fire regimes (Hempson *et al*., 2018; Steel *et al*., 2021; Wilkin *et al*., 2021). Additional diversity metrics have been developed that are designed to be both generalisable and ecologically relevant (Nowosad & Stepinski, 2019) and are beginning to be applied in fire ecology (Doherty *et al*., 2024). These are discussed further in Section IV.

##### (iv) Spatial heterogeneity of severity

Metrics of spatial variation in severity were frequently applied at a landscape scale to test how heterogeneity in burn severity influences habitat selection (Volkmann & Hodges, 2024) or species richness (i.e. testing the pyrodiversity hypothesis; Linley *et al*., 2024). A long‐term monitoring study of birds that coincided with a large unplanned fire found that most bird species responded negatively to the amount of severely burnt forest in the landscape (Lindenmayer *et al*., 2014), with large variation among species. Interestingly, the strength of the result was somewhat offset by strong site fidelity, likely influenced by environmental features such as elevation and topography. Other studies on birds (Watson *et al*., 2012), plants (Wilkin *et al*., 2021), mammals (Chia *et al*., 2015), reptiles (Nimmo *et al*., 2013), and invertebrates (Avitabile *et al*., 2015) also found influences of underlying environmental gradients and locality, making this a universal consideration in all spatial analyses. Studies of single‐species responses to spatial heterogeneity of fire severity found intra‐species sources of ecological variation. For example, a series of studies radio‐tracking the black‐backed woodpecker (*Picoides arcticus*) (Stillman *et al*., 2019, 2021, 2022) demonstrated that this post‐fire habitat specialist exhibits age‐dependent habitat selection with juveniles and adults selecting patches of different fire severity to track resources. Likewise, in bighorn sheep (*Ovis canadensis*), response to spatial variation in fire severity was found to be mediated by sex‐specific dispersal (Donovan *et al*., 2021). The relationship between ecosystem responses and severity indices varies widely between vegetation types and the taxa being studied (Keeley, 2009). It is therefore necessary to evaluate the relevance of any generalised severity index such as dNBR or composite burn indices to the ecological process of interest.

##### (v) Spatial heterogeneity of seasonality and ignition points

Few studies utilised metrics of spatial seasonality or ignition points, making it difficult to draw meaningful insights. Studies utilising metrics of spatial seasonality were commonly conducted in the tree savannas of northern Australia and consistently defined their metrics based on this fire‐prone biome and its early–late dry season dynamics (Davies *et al*., 2021; Griffiths, Garnett & Brook, 2015; Radford *et al*., 2021; Russell‐Smith, Edwards & Price, 2012). Each study provided evidence suggesting that late dry season fires were leading to impacts on longer‐maturing tree species, resource depletion for dependent fauna, and consequently large declines in populations of birds and small mammals (Russell‐Smith *et al*., 2012; Woinarski *et al*., 2011).

Few studies examined the ecological effects of the spatial pattern of ignition points. Ignition points, whilst being relevant to fire patterns, were not perceived as relevant to many ecological impacts. Studies used simulations to test how different management techniques influence fire size and impact on populations (Bradstock *et al*., 2005, 2006). The ignition patterns themselves were not found to be significant but the rate of ignition highlighted the trade‐offs required between management to reduce fire size *versus* reducing frequency. A recent review into prescribed fire highlighted the need to understand how ignition patterns affect fire behaviour and tree survival (Hiers *et al*., 2020). Although Hiers *et al*. (2020) distinguished between prescribed fire and wildfire, the need for a better understanding of how fuel moisture, vegetation type, and other micro‐conditions around ignition points resulted in variable ecological impacts suggests it is an important and potentially overlooked metric in fire ecology. Likewise, understanding the ecological effects of metrics of fire spread, including speed, expansion, and duration is an area for future research (Andela *et al*., 2019).

## IV. RECOMMENDATIONS

### (1) Consider the context when choosing a metric

As highlighted in the studies detailed in Sections II and III, many processes are driving the proliferation of descriptive metrics in fire ecology. Due to the inherently close relationships between spatial patterns of fire and ecological processes, it is crucial to examine fire metrics alongside additional metrics providing context to draw out complex responses to fire. This can be described through three levels of context: the data context, the landscape context, and the species context.

#### (a) Data context

**Fig. 5:**
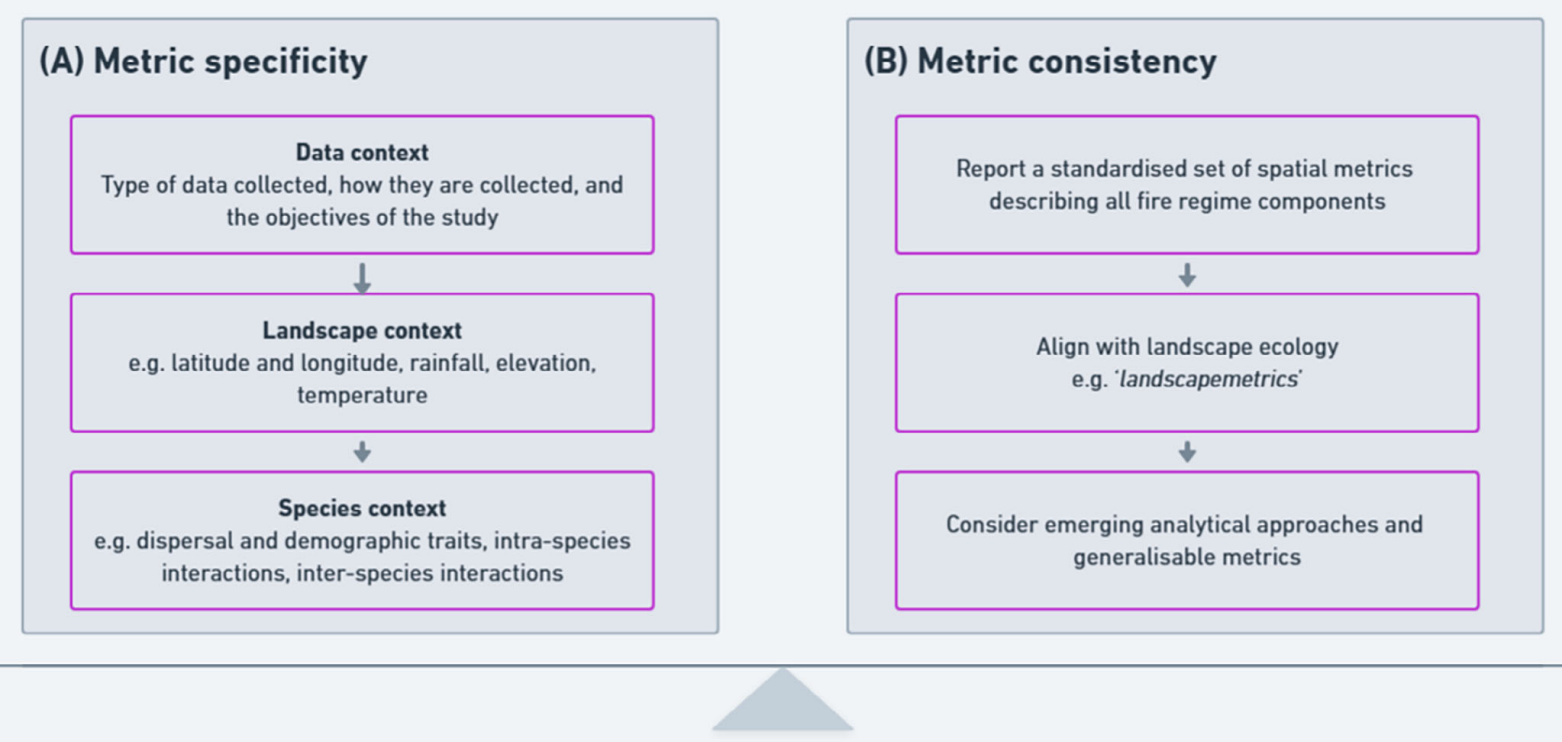
Recommendations for balancing (A) metric specificity and (B) metric consistency.

Key data considerations to be made before selecting spatial fire metrics include information on the type of data collected, how it is collected, and the objectives of the study (McGarigal, 1995; Fig. 5A). This information will define how the landscape is quantified and in doing so, will dictate the results and interpretations. Whether the objective is to understand how a single species' movements and habitat use are affected by fire, or to understand how landscape heterogeneity influences species richness, this objective will inform the scale and choice of metrics. For example, in fire ecology, the spatial fire data that come from remote sensing will define the size of the grain and the scale at which analyses can be undertaken. Technological advances have significantly reduced the spatial resolution of satellite imagery and allowed researchers to examine responses to fire at a finer scale (Szpakowski & Jensen, 2019). Despite the recognition of the importance of spatial scale, very few fire ecology studies utilise techniques such as scale optimisation to quantify relationships between fire and species habitat (Wan, Cushman & Ganey, 2020). Several studies included in this review examined species' responses to spatial fire metrics at multiple spatial scales and found that the scale of analysis is a crucial consideration (Avitabile *et al*., 2015; Farnsworth *et al*., 2014). We recommend that researchers consider how data, and methods to collect it, might influence the scale at which ecological responses to fire are measured and the effectiveness of chosen spatial metrics.

#### (b) Landscape context

Species' responses to fire can vary geographically as a result of variation in vegetation structure, climate, or ecological conditions (Nimmo *et al*., 2014). It is therefore essential to consider landscape context in fire ecology. Failure to consider landscape context adequately can lead to spurious results. For example, some studies attempting to test whether biodiversity and pyrodiversity are positively correlated have produced contrasting results with suggestions that fire regimes have a landscape context that is more important than a generalised rule (Jones & Tingley, 2022; Parr & Andersen, 2006). Within the studies examined in our review, some examples of landscape context variables include: temperature (Banks *et al*., 2017a; Kelly *et al*., 2017), topography (Bradstock *et al*., 2005), elevation (Lindenmayer *et al*., 2014), rainfall (Delaney *et al*., 2021), solar radiation (Haire & McGarigal, 2010), latitude and longitude (Taylor *et al*., 2012), wind direction (Bradstock *et al*., 2006), logging in the surrounding landscape (Lindenmayer *et al*., 2019), and winter severity (Wu *et al*., 1996). Vegetation structure has been shown to influence invertebrate responses to fire more directly than fire history (Brassard *et al*., 2023). These additional variables were frequently found to be important covariables explaining the geographically variable species response to fire and are therefore crucial to build an in‐depth landscape context for analyses.

#### (c) Species context

Trait variation within and among species will shape the fire response of individuals and species. Dispersal is a core consideration when choosing ecologically relevant spatial metrics, as many studies investigate whether a species' dispersal capacity moderates the negative effects of large, homogenous (and typically high‐intensity) fires on the population (Nimmo *et al*., 2019). Some studies examined in this review considered dispersal capacity by directly tracking the movement of individuals (Hope, 2012; Parker *et al*., 2021), including the dispersal mechanism or vector (Rocha *et al*., 2022), or measuring population dynamics (Banks *et al*., 2017a). Such approaches can reveal the spatially explicit population processes (such as dispersal and migration) that facilitate the recolonisation of sites after fire events, and hence population persistence through time. The outcomes of this research can lead to fire management recommendations that tailor spatial patterns of fire (e.g. amount of long unburnt habitat) to the dispersal capacity of target species (Legge *et al*., 2015; Robertson *et al*., 2022). Other studies in this review examined species traits including typical demographic rates (Bradstock *et al*., 2005), thermal tolerances (Robertson *et al*., 2022), territoriality and sociality (Lappan *et al*., 2021), and strategies for surviving the immediate and longer‐term effects of fire. Furthermore, interactions between species, including post‐fire competition (Coop & Schoettle, 2009), the presence of predators (Leahy *et al*., 2016), or the availability of prey (MacGregor *et al*., 2013), can shape species responses to fire.

### (2) Balance generalisable and specific metrics

Many fire regime components tend to be inherently highly correlated. For example, large fires, which may be more intense, burn a large proportion of the total area of a landscape and therefore contribute a disproportionate amount to mean fire interval (Gill & Allan, 2008; Li *et al*., 1999). Fire interval (or its inverse, fire frequency) is considered primary to fire size, as it significantly affects the vast majority of terrestrial species, as opposed to spatial fire variables being more important to species relying on dispersal to avoid the effects of individual fires (Gill & Allan, 2008). Despite this, spatially explicit variables are often necessary for studies looking at effects of fire, such as recolonisation by seed dispersal (Gill, Hoecker & Turner, 2021) or habitat use (Tomassini *et al*., 2024). The proliferation of spatial metrics through technological advances has allowed researchers to apply metrics of greater specificity, tailored to the research objectives. This increase in metric options has the potential to trade off generality for precision, making it difficult to integrate findings across studies. Therefore, it seems preferable there are ecologically based reasons for deconstructing fire size (or fire interval) into its more specific spatial parts. These reasons are not clearly articulated in many studies, or for many metrics captured by our review, largely because the technique for estimating the spatial metric was not clearly described. Making the reasons behind decisions explicit helps readers understand how these decisions may have affected the results. We recommend that researchers balance the choice of metric between generalisable metrics, to allow integration across studies, and specific metrics, to address the research questions accurately (Fig. 5B).

### (3) Report a standardised set of spatial metrics

To increase the consistency of spatial metrics used, we recommend a set of metrics that capture the complexity of fire patterns without losing the necessary precision to answer individual study questions. These metrics are scale independent in extent and resolution and therefore broadly comparable across studies. In addition to reporting standardised metrics for the focus question, it should be possible to report standardised spatial metrics of all components of contemporary fire regimes. As outlined above, a key challenge in fire ecology is the inherent variability of fire effects between locations, biomes, taxa, and methodology. Part of this variability could be explained by the additional aspects of the fire regime that were not the focus of the study. With satellite data now readily available, it is possible to obtain basic spatial metrics describing all components of contemporary fire regimes. Reporting standardised metrics of all fire regime components will enable future meta‐analyses and systematic reviews to quantify fire effects more easily and determine generalities among studies. Below we highlight the definition and data requirements to produce these metrics and ensure they are comparable across studies.

#### (a) Definition requirements

(1) Define the spatial and temporal boundaries of the study landscape, including the extent and resolution.
(2) Define what constitutes a fire event, according to the study objectives and ecosystem context, e.g. individual fires, or annual or seasonal burn patterns. Different definitions may be relevant in different systems; for example, a series of fires implemented as part of a prescribed burning program during one season could be considered a single event.
(3) Define fire severity categories, whether they were derived using a standard measure (e.g. composite burn indices) or custom categories.

#### (b) Data requirements

For most metrics, analyses only require rasters of burnt and unburnt cells per unit time to calculate metrics such as fire size, fire shape, spatiotemporal patterns, and spatial seasonality. Additional data representing fire severity or intensity classes per unit time are needed to assess spatial severity. For spread dynamics, high‐temporal‐resolution rasters (e.g. daily burn‐date data) are essential to capture the progression of fire fronts through time.

Other studies have similarly called for standardised metrics, however, they often have a more specific focus like pyrodiversity metrics (Jones & Tingley, 2022) or metrics of fire effects on populations or habitat (Haslem *et al*., 2024). Here, we encourage reporting metrics that broadly describe spatial patterns of the fire event and fire regime (Table 5).

**Table 5:** Recommended spatial metrics for standardised reporting in fire ecology.

Metric groups	Metrics	Units or range	Data requirements and notes on derivation	Example of use
Fire size	Area‐weighted mean of the area of individual fires	ha or km^2^	Requires the final area burned by each individual fire in a landscape (in a defined period of time). The area‐weighted mean accounts for the greater influence of large fires on overall landscape shape. Can be calculated within GIS software or *landscapemetrics* R package (e.g. AREA_CV, AREA_MN, AREA_SD).	Andela *et al*. (2019)
Fire shape	Area‐weighted mean fractal dimension index	1–2	Quantifies the geometric complexity of fire perimeters based on the perimeter–area relationship. Values approach 1 for simple, compact fires and 2 for highly irregular or fragmented fires. The area‐weighted mean accounts for the greater influence of large fires on overall landscape shape. Can be calculated using the *landscapemetrics* R package (e.g. FRAC_CV, FRAC_MN, FRAC_SD).	Rosu *et al*. (2024)
Spatiotemporal	Mean proportion of landscape burnt per unit time	0–1 proportion; or % year^−1^; or % decade^−1^	Requires the final area occupied by fires each year (or decade) in a landscape, extended across multiple years (or decades). This metric is related to the mean fire frequency (in units: fires year^−1^ or fires decade^−1^), and is the inverse of the mean fire interval. Can be calculated within GIS software.	Russell‐Smith *et al*. (1997)
	Temporal standard deviation (arcsine–sqrt transformed) in the proportion of the landscape burnt per unit time	radians	Captures temporal variability in the proportion of the landscape burnt. The arcsine–sqrt transformation stabilises variance and improves comparability and is applied to data prior to calculating the standard deviation.	Knapp & Keeley (2006)
	Spatial standard deviation (arcsine–sqrt transformed) in fire return interval among raster cells	radians	Captures spatial heterogeneity in fire frequency, indicating presence of fire refuges or hotspots in the landscape. The arcsine–sqrt transformation stabilises variance and improves comparability and is applied to data prior to calculating the standard deviation.	Knapp & Keeley (2006)
Spatial severity	Proportion of landscape burnt in specified severity (intensity) class	0–1 proportion or %	Requires the final area burned by fires and categorised by defined severity class. Can be calculated within GIS software or *landscapemetrics* R package (e.g. PLAND).	Reilly *et al*. (2017)
	Temporal standard deviation (arcsine–sqrt transformed) in mean fire severity across the landscape per unit time	radians	Captures temporal variability in fire severity, given a fire occurred in that cell in that year. The arcsine–sqrt transformation stabilises variance and improves comparability and is applied to data prior to calculating the standard deviation.	Miller & Safford (2012)
	Spatial standard deviation (arcsine–sqrt transformed) in mean fire severity among raster cells	radians	Captures spatial heterogeneity in fire severity, given a fire occurred in that cell in that year. The arcsine–sqrt transformation stabilises variance and improves comparability and is applied to data prior to calculating the standard deviation.	Miller & Safford (2012)
Spatial seasonality	Ratio of area burnt in a specified season to the total area burnt (over *X* years)	0–1	Requires the final area burned by each individual fire in a landscape and the time of year (e.g. month).	Radford *et al*. (2021)
Fire spread	Area‐weighted mean of fire speed	km day^−1^	The mean speed (i.e. rate of spread), expansion and duration of every individual fire in a landscape, in a set period of time. The area‐weighted mean accounts for the greater influence of large fires on overall landscape shape. Requires high‐temporal‐resolution rasters (e.g. daily burn‐date data).	Andela *et al*. (2019)
	Area‐weighted mean of fire expansion	km^−2^ day^−1^ or ha day^−1^		
	Area‐weighted mean of fire duration	days		

### (4) Align with landscape ecology

Fire ecology is a field of study that has strong overlap with landscape ecology. The metrics used in fire ecology are typically drawn from the broader literature and adapted in terminology and methodology to suit the specific needs of fire systems. Examining ecological processes using metrics consistent within the broader landscape literature would facilitate the integration of studies across both fields. Metrics to quantify landscape patterns proliferated throughout the 1980s and 1990s, and hundreds of measures have been proposed (Gustafson, 1998). Conveniently, many of these metrics or landscape indices were developed and collected into a database and software tool: FRAGSTATS (McGarigal, 1995). These metrics have been incorporated into the *landscapemetrics* R package (Hesselbarth *et al*., 2019) which contains over 100 metrics at three different scales (patch, class, and landscape) and encompasses edge, core, shape, aggregation, and diversity measurements. The use of the *landscapemetrics* R package may not fit with the methodology for all studies. However, referring to the database and metric definitions can help researchers find the most appropriate metric. Fire ecology methods and insights can also inform other fields such as landscape ecology, particularly around ways to deal with temporally complex patterns. Corry (2019) called for landscape ecologists to pay greater attention to the temporal dynamics of landscapes in much the same way that this review calls for fire ecologists to consider the spatial dynamics of fire regimes. With increasing data availability and advances in analytical capabilities, the divide between the two fields can be reduced, with lessons in spatial analysis flowing to fire ecology and lessons in temporal analysis flowing to landscape ecology. A recent review in landscape ecology emphasised the important role of computational methods in dealing with increasingly available data and high degree of complexity (Hesselbarth *et al*., 2025). Many of these methods also apply to fire ecology, some of which are discussed below.

### (5) Stay up to date with emerging metrics and techniques

To account for the proliferation of spatial metrics, new techniques and metrics are being developed that can measure entire landscapes accurately, considering their spatial complexity, whilst still being generalisable and ecologically relevant (Nowosad & Stepinski, 2019). Studies have found success in utilising Principal Components Analysis on a range of spatial metrics and using vectors consisting of the top principal components instead of the metrics themselves (Cushman, McGarigal & Neel, 2008; Nowosad & Stepinski, 2018). Using information theory, Nowosad & Stepinski (2019) derived five new metrics that outperform other techniques, which have been integrated into the *landscapemetrics* package in R (Hesselbarth *et al*., 2019). These metrics utilise spatial entropy, a modification of Shannon entropy, but extend it with information theory so that the resulting metrics are generalisable and ecologically relevant. The first two metrics listed below, (*H*(*y*) and *U*) can be reported across all fire regime components and the following three can be used where appropriate:

(1) *H*(*y*): marginal entropy, which reflects the diversity of fire classes (e.g. severity classes, fire age classes).
(2) *U*: relative mutual information, which reflects the predictability or clumpiness of spatial patterns.
(3) *H*(*y*|*x*): conditional entropy, quantifying configurational complexity or heterogeneity.
(4) *H*(*x*, *y*): joint entropy, capturing overall spatial complexity of the landscape.
(5) *I*(*y*, *x*): mutual information, measuring adjacency‐based predictability between fire classes.

These metrics can then be utilised in a broad range of analyses to lead the fire ecology field in a more coordinated direction. We found only one fire ecology study that has used an entropy metric. Doherty *et al*. (2024) applied the conditional entropy metric as a measure of burn patchiness, which accurately accounts for configurational complexity in highly heterogenous areas.

Other recently developed R packages that provide ways to analyse complex spatial metrics include *Motif* (Nowosad, 2021), which provides methods to describe and quantitatively compare spatial patterns for categorical raster data sets, and *Belg* (Nowosad & Gao, 2020), used for calculating Boltzmann entropy (i.e. landscape configurational entropy) of landscape mosaics and gradients. However, any newly developed metric needs to be tested rigorously to ensure it provides intuitive and useful results for fire ecology. Some complex metrics designed to summarise many aspects of landscape patterns turn out not to measure what is expected, whilst simple and direct metrics can be more useful in the long run (Martin *et al*., 2025).

New metrics and techniques are emerging to address inconsistencies within the fire ecology literature. Technological advances to improve consistency include using geostationary satellite data, which offers near‐continuous fire behaviour information and has been shown to improve correlations between intensity and severity metrics (Chatzopoulos‐Vouzoglanis *et al*., 2024). Further, machine learning is being used with Google Earth Engine data to map and predict fire effects (i.e. composite burn indices) that are ecologically relevant across large scales (Parks *et al*., 2019) and shows potential for global‐scale analyses.

## V. CONCLUSIONS

(1) As technological advances allow greater specification of spatial metrics in fire ecology, these advances have the potential to trade off generality for precision, which in turn may reduce comparability among studies.
(2) To ensure ecologically sound selection, definition, and application of spatial metrics, we recommend that fire ecologists consider: (*i*) providing context on the type of data, the way they are collected and the objectives of the investigations; (*ii*) providing context on the relevant landscape and habitat features; and (*iii*) providing context on the species' traits and ecological attributes. With these contexts in mind, fire ecologists will have a clearer understanding of the available metrics to accurately analyse their study system.
(3) By choosing the most generalisable metrics possible, we can ensure the fire ecology field utilises the strength of a cohesive scientific conversation. The complex problems of fire ecology will be better overcome, not by many metrics operating in isolation, but by careful ecological consideration, robust methodologies, and systematic improvements based on synthesised knowledge.

## AUTHOR CONTRIBUTIONS

This paper was conceived by S. C. B., G. J. C., and A. R. C. All authors contributed to the ongoing design. A. R. C. led the data curation, analysis, and writing. All authors provided advice on analysis and edited drafts of the manuscript.

## Supporting information


**Appendix S1.** Articles included in the topic modelling analysis and qualitative review.

**Fig. S1.** Number of studies included in the topic modelling analysis per year.
**Fig. S2**. Optimum number of topics identified within the fine ecology literature using the Deveaud2014 method.
**Table S1**. The probability of each topic across the entire corpus and the number of documents to which each topic is assigned as the primary topic.
**Table S2**. The 20 topics from the fire ecology literature with output from binomial generalised linear models (GLMs) showing the change in topic popularity between 1991 and 2025.
**Fig. S3**. Visualisation of trends in the whole corpus.
**Fig. S4**. The proportion of trends in each year that were allocated to each topic as the dominant topic, with all topics and fitted generalised linear models (GLMs) and generalised additive models (GAMs).
**Table S3**. Number of studies by country from the additional search query.

## Data Availability

The studies included in the topic modelling and qualitative review section of the paper are available in the online Supporting Information.
