# Supplementary material for: Spatial metrics in fire ecology: seeking consistency amidst complexity
Source: Biol Rev Camb Philos Soc. 2026 Feb 3;101(4):1641–61. doi: 10.1002/brv.70140 (PMC13326798; doi:10.1002/brv.70140)
Supplement: Supplementary file 2 — Fig. S1. Number of studies included in the topic modelling analysis per year. Fig. S2. Optimum number of topics identified within the fine ecology literature using the Deveaud2014 method. Table S1. The probability of each topic across the entire corpus and the number of documents to which each topic is assigned as the primary topic. Table S2. The 20 topics from the fire ecology literature with output from binomial generalised linear models (GLMs) showing the change in topic popularity between 1991 and 2025. Fig. S3. Visualisation of trends in the whole corpus. Fig. S4. The proportion of trends in each year that were allocated to each topic as the dominant topic, with all topics and fitted generalised linear models (GLMs) and generalised additive models (GAMs). Table S3. Number of studies by country from the additional search query. [file BRV-101-1641-s001.docx]

**Spatial metrics in fire ecology: seeking consistency amidst complexity**

**Supporting information**

**Fig. S1.** Number of studies included in the topic modelling analysis per year.


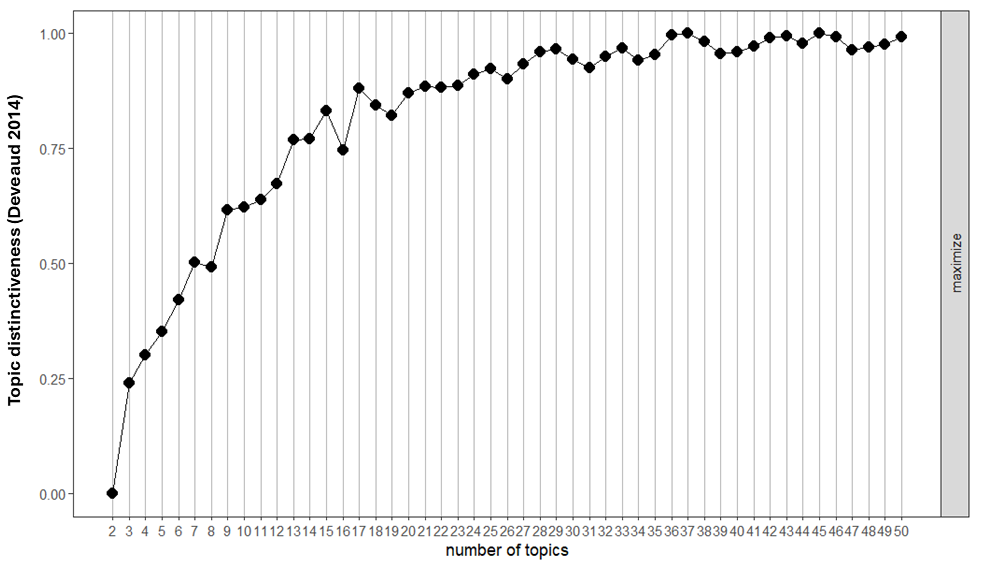


**Fig. S2.** Optimum number of topics identified within the fire ecology literature calculated using the Deveaud2014 method. 20 topics were chosen as this is after the elbow point when variation stabilises.

**Table S1.** The probability of each topic across the entire corpus and the number of documents to which each topic is assigned as the primary topic.

| Topic | Topic probability | Number of documents per topic |
| --- | --- | --- |
| Animal ecology | 0.049 | 496 |
| Climate change | 0.051 | 397 |
| Disturbance | 0.049 | 251 |
| Ecological response | 0.051 | 579 |
| Fire prediction | 0.050 | 363 |
| Forestry | 0.048 | 477 |
| Fuel treatment | 0.049 | 570 |
| Grassland | 0.049 | 525 |
| Management | 0.053 | 492 |
| Plant ecology | 0.050 | 657 |
| Post-fire recovery | 0.048 | 262 |
| Regional analysis | 0.053 | 401 |
| Remote sensing | 0.057 | 882 |
| Season | 0.048 | 272 |
| Severity | 0.050 | 207 |
| Soil ecology | 0.049 | 573 |
| Spatial | 0.050 | 275 |
| Temporal | 0.050 | 398 |
| Tree dynamics | 0.048 | 524 |
| Vegetation | 0.048 | 224 |

**Table S2.** The 20 topics from the fire ecology literature with output from binomial generalised linear models (GLMs) showing the change in topic popularity between 1991 and 2025. Significance is shown as * <0.05, ** <0.01, ***<0.001.

| Topic | Trend | Significance |
| --- | --- | --- |
| Animal ecology | Stable |  |
| Climate change | Increasing | *** |
| Disturbance | Increasing | *** |
| Ecological response | Decreasing | ** |
| Fire prediction | Stable |  |
| Forestry | Decreasing | *** |
| Fuel treatment | Stable |  |
| Grassland | Decreasing | *** |
| Management | Increasing | *** |
| Plant ecology | Decreasing | *** |
| Post-fire recovery | Stable |  |
| Regional analysis | Increasing | *** |
| Remote sensing | Increasing | *** |
| Season | Decreasing | *** |
| Severity | Stable |  |
| Soil ecology | Increasing | ** |
| Spatial | Decreasing | *** |
| Temporal | Decreasing | *** |
| Tree dynamics | Decreasing | *** |
| Vegetation | Decreasing | *** |


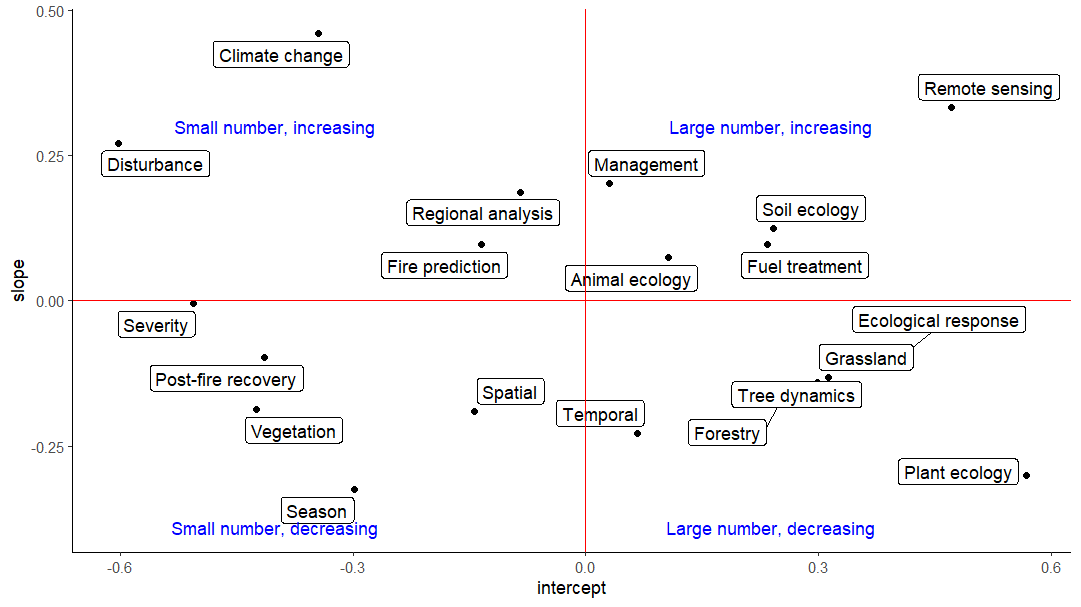


**Fig. S3.** Visualisation of trends in the whole corpus. We used a generalised linear mixed-effects model (GLMM) with a negative binomial distribution to examine the relationship between the number of documents published on each topic and year. The random intercept and slope for each topic were extracted from the model to represent topic-specific deviations in document counts over time. A positive random intercept indicated a higher-than-average number of documents published on that topic during the study period, while a positive slope indicated an increase in the number of documents published on that topic over time (see Fig. S1).


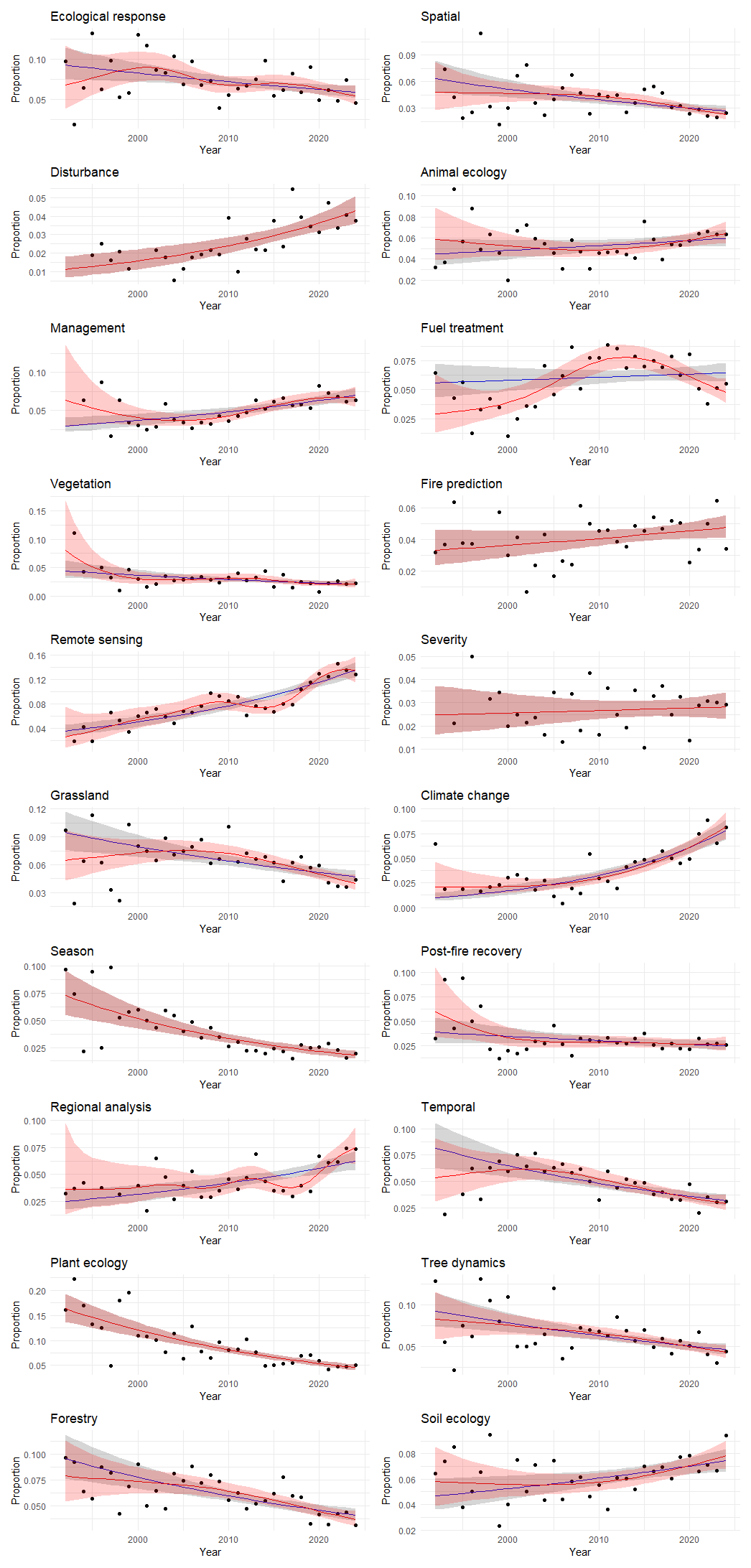


**Fig. S4.** The proportion of studies in each year that were allocated to each topic as the dominant topic, with fitted generalised linear models (GLMs; blue lines) and generalised additive models (GAMs; red lines) and 95% confidence intervals (shaded area).

**Table S3.** Number of studies by country from the additional search query. We conducted an additional database query to supplement the data set with studies specifically focussed on spatial fire metrics and their ecological effects. No specific timeframe, biome or taxonomic group was set to allow for the evaluation of trends in spatial fire metrics temporally, geographically and ecologically. Search terms were selected to maximise papers and maintain relevance using the Boolean query: (TI=(*Fire* OR Burn* OR Pyro*)) AND (ALL=(Spatial* OR Pattern* OR Mosaic* OR Matri* OR Patch* OR "Distance" OR size* OR shape* OR extent* OR frequenc* OR intensit* OR severit* OR "Ignition point*" OR Season* OR "Fire regime*" OR Occurrence* OR "Habitat use*" OR Diversit* OR Abundance* OR Movement* OR Dispers*)). This query resulted in 94,980 articles which were refined by relevant *Web of Science* categories (Ecology, Forestry, Environmental sciences, Remote sensing, Plant sciences, Biodiversity conservation, Biology, Ornithology, Environmental studies, and Soil science) and by excluding the Citation Topics Meso fields (composting, fracking, and ocean color). Using these filters, we extracted 21,999 articles to evaluate for relevance.

| Country | Count | % of total |
| --- | --- | --- |
| USA | 9,903 | 44.03 |
| Australia | 2,631 | 11.70 |
| Spain | 1,947 | 8.66 |
| China | 1,893 | 8.42 |
| Canada | 1,825 | 8.11 |
| Brazil | 1,026 | 4.56 |
| England | 932 | 4.14 |
| Germany | 900 | 4.00 |
| Italy | 819 | 3.64 |
| Portugal | 701 | 3.12 |
